# Association Between Picky Eating and Stunting Among Ethnic Minority Children Aged 12–35 Months in a Mountainous Area of Northern Vietnam: A Cross-Sectional Study

**DOI:** 10.3390/diseases14060183

**Published:** 2026-05-22

**Authors:** Thi Thu Ha Le, Thanh Hang Ngo, Thi Hoa Ho, Thi Thu Nguyen, Huu Chinh Nguyen, Thi Tu Quyen Bui, Thi Kieu Chinh Pham, Thi Thu Lieu Nguyen, Thi Huong Le

**Affiliations:** 1 Hanoi Medical University, Hanoi 100000, Vietnam; ltth@huph.edu.vn (T.T.H.L.); phamthikieuchinh@gmail.com (T.K.C.P.); lethihuong@hmu.edu.vn (T.H.L.); 2 Hanoi University of Public Health, Hanoi 100000, Vietnam; ngothanhhang293@gmail.com (T.H.N.); thu.ntt05dec@gmail.com (T.T.N.); btq@huph.edu.vn (T.T.Q.B.); 3 Institute of Nutrition Research and Development, Hanoi 100000, Vietnam; hohoa1905@gmail.com; 4 National Institute of Nutrition, Hanoi 100000, Vietnam; nguyenhuuchinhvdd@gmail.com; 5 Thai Binh University of Medicine and Pharmacy, Hungyen 17000, Vietnam

**Keywords:** selective eating behavior, linear growth faltering, zinc deficiency, mountainous region, feeding practices, cross-sectional study

## Abstract

Background: Stunting remains a major public health problem among ethnic minority children in mountainous areas of Vietnam. Picky eating has been suggested as a potential behavioral risk factor for poor child growth, but evidence from vulnerable rural populations remains limited. This study examined the association between picky eating and stunting among ethnic minority children aged 12–35 months in Vietnam. Methods: This cross-sectional study was conducted from October to November 2025 in two communes of Phu Tho province, formerly part of Lac Son District, Hoa Binh Province, Vietnam. A total of 341 children aged 12–35 months and their caregivers were included. Data were collected using structured interviewer-administered questionnaires on feeding practices and child characteristics. Picky eating was assessed based on caregiver-reported behaviors. Anthropometric measurements were performed according to standard procedures, and height-for-age Z-scores were calculated using the WHO Child Growth Standards. Zinc status was assessed in a subsample of children. Bivariate and multivariable logistic regression analyses were conducted to identify factors associated with stunting. Adjusted odds ratios (AORs) with 95% confidence intervals (95% CIs) were reported. Results: The prevalence of picky eating was 39.6%, while 24.9% of children were stunted. Zinc deficiency was identified in 41.9% of children with available blood samples. In multivariable analysis, picky eating was significantly associated with increased odds of stunting (AOR = 3.63; 95% CI: 1.71–7.70). Snacking before main meals was also independently associated with stunting (AOR = 1.81; 95% CI: 1.01–3.24). In contrast, zinc deficiency was associated with stunting in crude analysis but was not statistically significant after adjustment. Other factors, including child age, sex, caregiver identity, and timing of complementary feeding, were not independently associated with stunting. Conclusions: Picky eating was common and was independently associated with stunting among ethnic minority children in this mountainous setting. These findings suggest that behavioral feeding practices, particularly picky eating and pre-meal snacking, warrant attention in nutritional programs targeting this population; however, longitudinal studies are needed to confirm the direction of this relationship.

## 1. Introduction

Stunting remains a major indicator of chronic undernutrition, leading to severe long-term consequences for child development, survival, and intergenerational health inequities [1,2,3]. According to the WHO, globally, there are 150.2 million children under 5 affected by stunting and 42.8 million children under 5 suffering from wasting, of which 12.2 million have severe wasting [1]. Children aged 12–35 months are particularly vulnerable to growth faltering due to rapid physical growth, high nutrient requirements, and the critical transition from milk feeding to family foods [4]. According to the Vietnamese General Nutrition Survey 2020 report, Vietnam’s nutritional situation has shown significant improvement. The low malnutrition rate among children under 5 years old is 19.6%, which is classified as medium risk by the WHO [5]. However, this rate remains very high in rural and mountainous areas, at over 30%, and is highest among children aged 12–35 months. The Northern Mountainous region currently reports the highest stunting prevalence nationwide at 37.4% [5]. Furthermore, ethnic minority children experience stunting rates up to twice as high as those of the Kinh majority, with approximately one in three ethnic minority children suffering from chronic malnutrition [6]. Recent national data also highlight that among children aged 6–23 months, only 36.7% achieved the minimum dietary diversity, exacerbating the risk of growth faltering in disadvantaged settings [7]. Concurrently, this developmental window often marks the emergence of picky eating, a selective behavior characterized by persistent refusal of certain foods, limited dietary variety, and problematic mealtime behaviors. Globally, meta-analyses estimate the prevalence of childhood picky eating to be around 22%, though reports vary widely from 13% to over 50% depending on the population and assessment criteria [8,9,10]. While international evidence suggests that picky eating may adversely affect nutritional status, findings remain inconsistent across different populations and study designs [10,11,12,13]. Current evidence on picky eating in Vietnam has largely focused on urban or general populations. Studies conducted in major cities such as Hanoi, Ho Chi Minh City, and Hue report that picky eating prevalence ranges from 22% to over 54% among preschool-aged children [14,15,16]. However, these studies do not address whether problematic eating behaviors directly drive chronic growth faltering in rural areas. Specifically, the prevalence and impact of picky eating on stunting among highly vulnerable ethnic minority toddlers in mountainous settings remain completely unexplored. Ethnic minority children in mountainous Vietnam face a distinct constellation of risk factors that may amplify the nutritional consequences of picky eating, including geographic isolation, limited household food diversity, reliance on monotonous staple-based diets with low micronutrient density, and limited caregiver knowledge of age-appropriate feeding practices. In this context, picky eating may carry more severe nutritional consequences than in urban settings with greater food availability and access to nutrition services, making it a particularly important behavioral risk factor in these vulnerable communities.

Addressing this critical gap, the present study aimed to examine the association between picky eating and stunting among ethnic minority children aged 12–35 months in a mountainous area of northern Vietnam. We hypothesized that children exhibiting picky eating behaviors would have significantly higher odds of stunting than those without such behaviors.

## 2. Materials and Methods

### 2.1. Study Design and Setting

A cross-sectional study was conducted from October to November 2025 in Muong Vang and Nhan Nghia communes, in Phu Tho Province, formerly belonging to Lac Son District, Hoa Binh Province, Vietnam. These communes were selected because of their persistently high burden of child malnutrition, challenging socioeconomic conditions, and the absence of active nutrition intervention programs at the time of data collection.

### 2.2. Study Participants

The study population comprised children aged 12–35 months and their mothers residing in the two selected communes. This age range was selected for three reasons: (i) it represents the highest-risk window for picky eating and stunting, as cumulative nutritional deficits from inadequate dietary intake begin to manifest as chronic growth faltering from 12 months onward; (ii) children aged 36 months and above typically attend preschool or kindergarten where institutionally provided meals introduce additional confounders for the assessment of home-based feeding practices; and (iii) 35 completed months is the last month before the third birthday, consistent with WHO completed-month age conventions. Eligible participants were children living in the study area during the data collection period whose mothers agreed to participate. Children with congenital disorders or those experiencing acute or chronic illness at the time of data collection were excluded.

### 2.3. Sample Size

The required sample size was calculated using the single population proportion formula with a 95% confidence level (Z_{1 − α/2} = 1.96), a relative error (ε = 0.2), and an estimated stunting prevalence of 23.5% [17]. The minimum required sample size was 313 children. After allowing for a 5% non-response rate, the final target sample size was 329 children.

### 2.4. Sampling Procedure

The two selected communes were purposively chosen because of their challenging socioeconomic conditions and high burden of child malnutrition. A complete list of children aged 12–35 months residing in the two communes was obtained from the commune health station registries of Muong Vang and Nhan Nghia, comprising 502 children in total. All children on the list were invited to participate through direct outreach conducted by commune health station staff and village health workers. Of these, 341 children who met the eligibility criteria and whose caregivers provided voluntary informed consent were enrolled, exceeding the minimum required sample size of 329 children calculated a priori.

### 2.5. Data Collection

Data were collected through face-to-face interviews with mothers using a structured interviewer-administered questionnaire. The questionnaire collected information on child, maternal, and household characteristics, including child age, sex, birth weight, breastfeeding history, complementary feeding practices, maternal age, education, ethnicity, household size, and household economic status.

### 2.6. Assessment of Nutritional Status

Trained data collectors performed anthropometric measurements following standardized procedures. Body weight was measured using a calibrated scale to the nearest 0.1 kg. Recumbent length was measured for children aged < 24 months, and standing height was measured for those aged ≥ 24 months, to the nearest 0.1 cm using standard anthropometric equipment in accordance with WHO procedures. Height-for-age z-scores (HAZ) were calculated using the World Health Organization (WHO) Child Growth Standards 2006 and the WHO Anthro software version 3.2.2. Stunting was defined as HAZ < −2 standard deviations [18].

### 2.7. Assessment of Zinc Status

Biochemical measurement: Serum zinc concentration (µg/dL) was measured using standard laboratory procedures. Serum zinc concentration was interpreted using IZiNCG-recommended, sampling-condition-specific cutoffs. In children aged < 10 years, zinc deficiency is defined as a serum zinc level < 65 µg/dL in morning non-fasting samples.

### 2.8. Assessment of Picky Eating

Picky eating, defined as a selective eating behavior characterized by persistent refusal of certain foods, limited dietary variety, and problematic mealtime behaviors, was assessed using a caregiver-reported questionnaire adapted from a standardized, previously validated tool developed by Hoang Thi Bach Yen [19]. The instrument comprises 8 items assessing three domains: food refusal (e.g., refusing specific foods or entire food groups), limited dietary variety, and problematic mealtime behaviors (e.g., prolonged meal duration, requiring distraction during meals). Each item is scored on a 3-point scale (0–3), yielding a total score of 0–24. Children with a total score exceeding 12 and symptom duration of at least one month were classified as having picky eating, in accordance with the predefined criteria established in the original validation study. The tool has previously been validated in Vietnamese children with a Cronbach’s alpha coefficient of 0.878 [19]. Prior to the main data collection, the instrument was pilot-tested in the study communes to assess its comprehensibility and applicability among the local ethnic minority population, and necessary clarifications were made to ensure cultural and linguistic appropriateness.

### 2.9. Study Variables

The primary outcome variable was stunting status, categorized as stunted or non-stunted. The primary exposure variable was picky eating. Other covariates included child characteristics, maternal characteristics, household factors, complementary feeding practices, and zinc status.

### 2.10. Data Analysis

Data were entered into EpiData and checked for completeness and consistency before analysis using SPSS version 27. Descriptive statistics were used to summarize participant characteristics. Continuous variables were presented as means and standard deviations, whereas categorical variables were presented as frequencies and percentages. Bivariate analyses were performed to examine the association between each independent variable and stunting status using the chi-square test for categorical variables and the independent-samples t-test for continuous variables, as appropriate. Variables were selected for the multivariable logistic regression model using a two-step approach. First, all variables with *p* < 0.20 in bivariate analysis were considered as candidates. Second, variables with established epidemiological relevance to stunting including child age, sex, birth weight, exclusive breastfeeding, maternal education, household economic status, and zinc deficiency were additionally considered regardless of their bivariate *p*-value. Given 85 stunting events, the multivariable model was restricted to maintain an events-per-variable ratio of approximately 10 and to avoid overfitting. Variables that did not reach *p* < 0.20 in bivariate analysis and lacked strong a priori epidemiological justification were excluded from the final model. Crude odds ratios (ORs), adjusted odds ratios (AORs), and 95% confidence intervals (CIs) were reported. Statistical significance was defined as a two-sided *p*-value < 0.05.

### 2.11. Ethical Considerations

Ethical approval for the study was obtained from the Institutional Review Board of Hanoi University of Public Health (Decision No. 404/2025/YTCC-HD3). The study was conducted in accordance with the Declaration of Helsinki. All study procedures were explained to the participants before data collection. Written informed consent was obtained from all participating mothers/caregivers before enrollment. Participation was voluntary, and confidentiality of the collected information was strictly maintained throughout the study.

## 3. Results

Table 1 summarizes the sociodemographic characteristics of the 341 participating children and their caregivers. More than half of the children were aged 24–35 months (55.7%), with a mean age of 24.8 ± 6.7 months. The majority were of Muong ethnicity (91.8%); 5.3% had low birth weight. Only one-third of children were exclusively breastfed (33.7%). Mothers were the primary caregivers for most children (66.0%). More than half of mothers had a high school education or above (52.2%), and most households were classified as having medium income (75.1%).

Table 2 shows that the prevalence of picky eating was 39.6% in the study population. Overall, 24.9% of children were stunted, including 17.6% with moderate stunting and 7.3% with severe stunting. Among the 334 children with available blood samples, 41.9% were zinc-deficient. Female children had slightly higher prevalences of picky eating and zinc deficiency than male children, whereas severe stunting was more common among males. Overall, the sex differences were small.

Table 3 shows the distribution of stunting according to picky eating status and selected feeding-related factors among children aged 12–35 months. The prevalence of stunting was significantly higher among picky eaters than among non-picky eaters (40.7% vs. 14.6%, *p* < 0.001). Stunting was also more common among children who snacked before main meals compared with those who did not (31.0% vs. 17.5%, *p* = 0.004), and among those whose meal duration exceeded 30 min compared with those whose meals lasted 30 min or less (31.9% vs. 21.2%, *p* = 0.029). No statistically significant differences in stunting were observed according to primary caregiver (*p* = 0.194), caregiver pressure to eat (*p* = 0.463), eating while watching television/mobile phone (*p* = 0.096), or age at initiation of complementary feeding (*p* = 0.760).

Table 4 presents the results of the crude and adjusted logistic regression analyses of factors associated with stunting among children aged 12–35 months. In the crude analysis, picky eating, zinc deficiency, snacking before main meals, and meal duration longer than 30 min were associated with higher odds of stunting. After adjustment, picky eating remained significantly associated with stunting (AOR = 3.625; 95% CI: 1.706–7.702; *p* < 0.001). (AOR = 3.625; 95% CI: 1.706–7.702). Snacking before main meals was also independently associated with stunting (AOR = 1.807; 95% CI: 1.007–3.243). In contrast, the association between zinc deficiency and stunting was attenuated after adjustment and was no longer statistically significant (AOR = 0.974; 95% CI: 0.475–1.998). Likewise, child age group, sex, birth weight, maternal education, household economic status, and meal duration > 30 min were not significantly associated with stunting in the adjusted model.

## 4. Discussion

This study found a substantial burden of nutritional and feeding-related problems among ethnic minority children aged 12–35 months in mountainous area of Northern Vietnam, including a stunting prevalence of 24.9%, zinc deficiency of 41.9%, and picky eating of 39.6%. The 24.9% stunting prevalence exceeds the Vietnamese national average of 19.6% for children under five, yet aligns closely with the elevated stunting rates (approximately 26.4%) persistently observed among ethnic minority populations in rural and mountainous regions [20]. Zinc deficiency remained common; the prevalence in this study was lower than the national estimate of 53.3% reported in the 2019–2020 General Nutrition Survey, suggesting that local Muong dietary practices or environmental factors may offer only marginal protective effects compared to other high-risk rural cohorts. Cultural and ethnic factors may also modulate picky eating behaviors and their nutritional consequences. Muong dietary practices, traditional food taboos, and caregiver feeding beliefs likely differ from those of urban Kinh populations, potentially influencing both the prevalence of selective eating and the range of nutritionally adequate foods available when children refuse certain items. Cross-ethnic comparative studies using standardized tools are needed to disentangle the roles of ethnicity, culture, and socioeconomic context in determining picky eating and its consequences for child growth. The 39.6% prevalence of picky eating falls consistently within the expected global estimate of 25% to 60% for early childhood [21]. Although overall sex differences were small, our data revealed several nuanced variations. Females exhibited slightly higher rates of picky eating (42.4% vs. 37.6%) and zinc deficiency (45.5% vs. 39.3%), whereas males were more susceptible to severe stunting (8.6% vs. 5.6%). These findings may suggest sex-specific metabolic demands and potential intra-household differences in food allocation, warranting further investigation. Together, these findings suggest that child growth in this setting may be influenced by both micronutrient inadequacy and suboptimal feeding behaviors.

A key finding of this study was the independent association between picky eating and stunting. Children with picky eating had significantly higher odds of being stunted after adjustment for potential confounders (*p* < 0.001; AOR = 3.625). This finding is consistent with the hypothesis that may reduce dietary variety and limit the intake of protein, zinc, iron, and other nutrients required for linear growth. Persistent food refusal in early childhood may therefore be associated with cumulative nutritional deficits over time. This interpretation is consistent with previous evidence showing that selective eating and food aversion may adversely affect dietary adequacy and later growth trajectories [22,23]. This finding should be interpreted cautiously, given the cross-sectional design; the direction and causality of this association cannot be established from these data. Snacking before main meals was also independently associated with stunting (AOR = 1.807). One possible explanation is that pre-meal snacking reduces hunger at regular mealtimes, which may lower the intake of staple foods and other nutrient-rich foods consumed during main meals, as recommended against in the WHO complementary feeding guidelines [4]. In settings where family meals provide the main source of daily nutrients, such a pattern may be associated with poorer dietary adequacy. Although prolonged mealtimes were significant only in bivariate analysis, this factor may still reflect feeding difficulties or poor appetite regulation and warrants further investigation.

An additional finding was that zinc deficiency was associated with stunting in crude analysis, but lost statistical significance after adjustment. This attenuation may suggest that the relationship between zinc deficiency and stunting is partly explained by other correlated behavioral or nutritional factors, particularly picky eating. Zinc deficiency may be associated with reduced appetite or altered taste perception, thereby increasing food refusal; however, this interpretation remains speculative and cannot be confirmed using cross-sectional data. Reverse causation is also possible, whereby children with chronic undernutrition or poor appetite are more likely to have both inadequate dietary intake and lower zinc status. Nevertheless, we cannot exclude the possibility that the zinc subsample (*n* = 334) lacked sufficient statistical power to detect a modest independent effect of zinc deficiency after adjustment for multiple covariates. Future studies with larger samples and objective dietary zinc intake measures are needed to clarify the independent contribution of zinc deficiency to stunting in this population.

To our knowledge, this is among the first studies to examine the association between picky eating and stunting specifically among ethnic minority toddlers in a mountainous district of Vietnam, a population underrepresented in the existing literature. The findings highlight that behavioral dimensions of feeding, particularly selective eating and pre-meal snacking, may be as important as micronutrient status in determining growth outcomes in this setting. Clinically, these findings suggest that nutritional interventions may need to go beyond mere caloric supplementation. Public health strategies should simultaneously address micronutrient deficiencies, including zinc supplementation where indicated, and integrate behavioral responsive feeding counseling. Beyond individual behavioral factors, structural determinants including household food insecurity, dietary diversity, and caregiver feeding knowledge likely interact with picky eating to shape growth outcomes in this setting. In mountainous ethnic minority communities, geographic isolation and reliance on subsistence agriculture constrain household food availability, potentially compounding the nutritional impact of picky eating. Limited caregiver knowledge of responsive feeding practices may further reduce caregivers’ capacity to manage picky eating behaviors effectively, particularly during the critical complementary feeding window. Nutritional programs should actively involve extended family caregivers and provide practical guidance on reducing pre-meal snacking to support adequate nutrient intake at main meals. Furthermore, reverse causation cannot be excluded: children with pre-existing growth faltering may have reduced appetite or altered sensory responsiveness, which could manifest as picky eating behaviors rather than being a contributing factor to poor growth. Longitudinal or interventional study designs are required to establish the temporal sequence of this association. Additionally, picky eating was assessed using a caregiver-reported behavioral tool rather than objective dietary intake methods such as 24 h dietary recall, food frequency questionnaires, or dietary diversity scores. Consequently, whether picky eating in this sample was associated with objectively inadequate macro- or micronutrient intake could not be confirmed. Future studies should integrate quantitative dietary assessment alongside behavioral screening tools to elucidate the nutritional pathway linking selective eating to growth outcomes.

Despite providing valuable localized epidemiological insights, this study has several limitations. The primary constraint is its cross-sectional design, which precludes definitively establishing temporal causality, specifically, whether maladaptive feeding behaviors directly cause stunting, or if pre-existing metabolic and zinc deficits in stunted children chronically suppress their appetite. Additionally, the retrospective collection of neonatal data may introduce recall bias. Socioeconomic status was captured using a three-category household income classification, which may not adequately reflect the multidimensional nature of poverty in mountainous communities, including food access constraints, agricultural seasonality, caregiving capacity, and geographic barriers to health services. Future studies should incorporate multidimensional measures of food security and household livelihood alongside income-based classifications. Furthermore, the homogeneous sample from a single district restricts the generalizability of these findings to broader ethnic minority populations in Vietnam. The study was conducted in two purposively selected communes in Hoa Binh Province, chosen for their high burden of child malnutrition and challenging socioeconomic conditions. While this selection enhances internal validity by focusing on a high-risk population, it limits the generalizability of findings to other ethnic minority communities with different geographic, cultural, or socioeconomic characteristics. Caution should therefore be exercised when extrapolating these results to broader ethnic minority populations across Vietnam’s Northern Mountainous region. Future randomized controlled trials focusing on behavioral feeding modifications paired with precise metabolic tracking, as well as prospective cohort studies incorporating quantitative dietary diversity scoring, are essential to fully elucidate the etiology of stunting in these demographics.

## 5. Conclusions

Picky eating is common and independently associated with stunting among children aged 12–35 months in this ethnic minority population. Behavioral feeding practices, particularly picky eating and pre-meal snacking, were associated with higher odds of stunting and warrant attention in nutritional programs targeting this population. These findings are associational in nature; longitudinal studies are needed to establish the temporal sequence of this relationship and to inform the design of targeted behavioral feeding interventions.

## Figures and Tables

**Table 1:** Sociodemographic characteristics of children aged 12–35 months and their caregivers (*n* = 341).

Characteristics	Categories	*n*	%
Child age (months)	12–23	151	44.3
	24–35	190	55.7
	Mean ± SD	24.8 ± 6.7	
Ethnicity	Muong	313	91.8
	Kinh	9	2.6
	Other	19	5.6
Birth weight	<2500 g	18	5.3
	≥2500 g	323	94.7
Exclusive breastfeeding	Yes	115	33.7
	No	226	66.3
Primary caregiver	Mother	225	66.0
	Grandparent/other	116	34.0
Maternal education	Primary or less	21	6.2
	Secondary	142	41.6
	High school or above	178	52.2
Household income	Poor	33	9.7
	Near poor	52	15.2
	Medium	256	75.1

**Table 2:** Prevalence of picky eating, zinc deficiency, and stunting among ethnic minority children aged 12–35 months.

Indicator		Male *n* (%)	Female *n* (%)	Total *n* (%)
Picky eating status	Picky eating	74 (37.6)	61 (42.4)	135 (39.6)
	Non-picky eating	123 (62.4)	83 (57.6)	206 (60.4)
Stunting status	Normal	147 (74.6)	109 (75.7)	256 (75.1)
	Moderate stunting	33 (16.8)	27 (18.8)	60 (17.6)
	Severe stunting	17 (8.6)	8 (5.6)	25 (7.3)
Zinc deficiency ^a^ (*n* = 334)	Yes	75 (39.3)	65 (45.5)	140 (41.9)
	No	116 (60.7)	78 (54.5)	194 (58.1)

^a^ Percentages for zinc deficiency were calculated among 334 children with available blood samples; 7 children did not participate in blood collection.

**Table 3:** Relationship between picky eating status and selected feeding practices with stunting of children in the study (n = 341).

Indicator		Stunted *n* (%)	Non-Stunted *n* (%)	Total *n* (%)	*p*-Value
Picky eating status	Picky eating	55 (40.7)	80 (59.3)	135 (39.6)	<0.001
	Non-picky eating	30 (14.6)	176 (85.4)	206 (60.4)	
Primary caregiver	Mother	61 (27.1)	164 (72.9)	225 (66.0)	0.194
	Grandparent/other	24 (20.7)	92 (79.3)	116 (34.0)	
Snacking before main meals	Yes	58 (31.0)	129 (69.0)	187 (54.8)	0.004
	No	27 (17.5)	127 (82.5)	154 (45.2)	
Meal duration > 30 min	Yes	38 (31.9)	81 (68.1)	119 (34.9)	0.029
	No	47 (21.2)	175 (78.8)	222 (65.1)	
Pressure to eat	Yes	35 (27.1)	94 (72.9)	129 (37.8)	0.463
	No	50 (23.6)	162 (76.4)	212 (62.2)	
Eating while watching television/phone	Yes	41 (21.5)	150 (78.5)	191 (56.0)	0.096
	No	44 (29.3)	106 (70.7)	150 (44.0)	
Age at initiation of complementary feeding	<6 months	15 (23.4)	49 (76.6)	64 (18.8)	0.760
	≥6 months	70 (25.3)	207 (74.7)	277 (81.2)	

Note: Data are presented as numbers (% row). *p*-values were derived from chi-square tests.

**Table 4:** Factors associated with stunting among children by logistic regression analyses.

Variable	Stunted *n* (%)	Non-Stunted *n* (%)	Crude OR (95% CI)	Adjusted OR (95% CI)
Picky eating				
Yes	55 (40.7)	80 (59.3)	4.033 (2.404–6.767)	3.625 (1.706–7.702)
No	30 (14.6)	176 (85.4)	1.00	1.00
Zinc deficiency ^a^				
Yes	49 (35.0)	91 (65.0)	2.446 (1.477–4.051)	0.974 (0.475–1.998)
No	35 (18.0)	159 (82.0)	1.00	1.00
Child age group (months)				
24–35	45 (23.7)	145 (76.3)	0.861 (0.526–1.409)	0.744 (0.427–1.297)
12–23	40 (26.5)	111 (73.5)	1.00	1.00
Sex				
Male	50 (25.4)	147 (74.6)	1.059 (0.644–1.743)	1.197 (0.693–2.068)
Female	35 (24.3)	109 (75.7)	1.00	1.00
Birth weight				
<2500 g	7 (38.9)	11 (61.1)	1.999 (0.749–5.333)	1.354 (0.447–4.101)
≥2500 g	78 (24.1)	245 (75.9)	1.00	1.00
Exclusive breastfeeding				
No	56 (24.8)	170 (75.2)	0.977 (0.582–1.64)	
Yes	29 (25.2)	86 (74.8)	1.00	-
Maternal education				
Secondary school or lower	47 (28.8)	116 (71.2)	1.493 (0.911–2.445)	1.541 (0.9–2.64)
High school or above	38 (21.3)	140 (78.7)	1.00	1.00
Household economic status				
Poor/Near poor	24 (28.2)	61 (71.8)	1.258 (0.724–2.186)	1.229 (0.66–2.29)
Medium	61 (23.8)	195 (76.2)	1.00	1.00
Primary caregiver				
Grandparent/other	24 (20.7)	92 (79.3)	0.701 (0.41–1.2)	
Mother	61 (27.1)	164 (72.9)	1.00	-
Snacking before main meals				
Yes	58 (31.0)	129 (69.0)	2.115 (1.26–3.551)	1.807 (1.007–3.243)
No	27 (17.5)	127 (82.5)	1.00	1.00
Meal duration > 30 min				
Yes	38 (31.9)	81 (68.1)	1.747 (1.057–2.886)	0.991 (0.549–1.79)
No	47 (21.2)	175 (78.8)	1.00	1.00
Pressure to eat				
Yes	35 (27.1)	94 (72.9)	1.206 (0.731–1.991)	
No	50 (23.6)	162 (76.4)	1.00	-
Eating while watching television/phone				
Yes	41 (21.5)	150 (78.5)	0.658 (0.402–1.078)	
No	44 (29.3)	106 (70.7)	1.00	-

^a^ Percentages for zinc deficiency were calculated among 334 children with available blood samples; 7 children did not participate in blood collection.

## Data Availability

The datasets analyzed during the current study are not publicly available because they contain individual-level information from child participants and caregivers that could compromise participant confidentiality. De-identified data are available from the corresponding author on reasonable request and subject to approval by the institutional ethics committee.
